# Neuroprotective effects of ex vivo-expanded regulatory T cells on trimethyltin-induced neurodegeneration in mice

**DOI:** 10.1186/s12974-022-02512-z

**Published:** 2022-06-11

**Authors:** Seon-Young Park, HyeJin Yang, Minsook Ye, Xiao Liu, Insop Shim, Young-Tae Chang, Hyunsu Bae

**Affiliations:** 1 Department of Physiology, College of Korean Medicine, Kyung Hee University, Seoul, 02453 South Korea; 2 Department of Biomedicine & Health Sciences, College of Medicine, The Catholic University of Korea, Seoul, 06591 South Korea; 3 Department of Chemistry, Pohang University of Science and Technology, Pohang, 37673 South Korea; 4 Department of Physiology, College of Medicine, Kyung Hee University, Seoul, 02453 South Korea; 5 Center for Self-Assembly and Complexity, Institute for Basic Science (IBS), Pohang, 37673 South Korea

**Keywords:** Regulatory T cells, Hippocampal neurodegeneration, Trimethyltin, Cell therapy, Microglia

## Abstract

**Background:**

Trimethyltin (TMT) is a potent neurotoxicant that leads to hippocampal neurodegeneration. Regulatory T cells (Tregs) play an important role in maintaining the immune balance in the central nervous system (CNS), but their activities are impaired in neurodegenerative diseases. In this study, we aimed to determine whether adoptive transfer of Tregs, as a living drug, ameliorates hippocampal neurodegeneration in TMT-intoxicated mice.

**Methods:**

CD4^+^CD25^+^ Tregs were expanded in vitro and adoptively transferred to TMT-treated mice. First, we explored the effects of Tregs on behavioral deficits using the Morris water maze and elevated plus maze tests. Biomarkers related to memory formation, such as cAMP response element-binding protein (CREB), protein kinase C (PKC), neuronal nuclear protein (NeuN), nerve growth factor (NGF), and ionized calcium binding adaptor molecule 1 (Iba1) in the hippocampus were examined by immunohistochemistry after killing the mouse. To investigate the neuroinflammatory responses, the polarization status of microglia was examined in vivo and in vitro using real-time reverse transcription polymerase chain reaction (rtPCR) and Enzyme-linked immunosorbent assay (ELISA). Additionally, the inhibitory effects of Tregs on TMT-induced microglial activation were examined using time-lapse live imaging in vitro with an activation-specific fluorescence probe, CDr20.

**Results:**

Adoptive transfer of Tregs improved spatial learning and memory functions and reduced anxiety in TMT-intoxicated mice. Additionally, adoptive transfer of Tregs reduced neuronal loss and recovered the expression of neurogenesis enhancing molecules in the hippocampi of TMT-intoxicated mice. In particular, Tregs inhibited microglial activation and pro-inflammatory cytokine release in the hippocampi of TMT-intoxicated mice. The inhibitory effects of TMT were also confirmed via in vitro live time-lapse imaging in a Treg/microglia co-culture system.

**Conclusions:**

These data suggest that adoptive transfer of Tregs ameliorates disease progression in TMT-induced neurodegeneration by promoting neurogenesis and modulating microglial activation and polarization.

## Background

The hippocampus, an area under the medial temporal lobe of the mammalian brain, plays a pivotal role in the neurobiology of learning and memory. It is one of the first regions damaged in Alzheimer’s disease (AD) [1, 2]. Hippocampal neurodegeneration accounts for the cognitive impairments observed in neurodegenerative disorders, such as AD [3]. There is a clinical association between hippocampal neurogenesis and cognition and microglia are important effectors of hippocampal neurogenesis. Activated pro-inflammatory microglia have a negative effect on hippocampal neurogenesis and cognitive processes [4].

Trimethyltin (TMT) is an organotin compound that is considered a potent neurotoxicant and causes behavioral alterations as well as learning and memory impairment in mammals [5, 6]. Cognitive impairment, including memory loss and learning impairment, developed in experimental animals exposed to TMT, indicating severe hippocampal damage [7]. It was reported that the levels of activated microglia and pro-inflammatory factors, such as TNFα, IL-1β, and NO were elevated in the hippocampus prior to neuronal death by TMT treatment in rodents. Consistently, previous studies have indicated that microglial activation by TMT exacerbates neuronal death in vivo and in vitro [8–10].

Regulatory T cells (Tregs) act as immune suppressors, playing a role in self-tolerance and immune homeostasis. Immune balance in the central nervous system (CNS) is tightly controlled by Tregs. Previous studies suggested that the Tregs suppress the microglial inflammation by promoting polarization toward anti-inflammatory M2 rather than pro-inflammatory M1 phenotype [11–13]. However, the suppressive activity of Tregs is dysregulated in neurodegenerative diseases, leading to neuroinflammation in these diseases. For these reasons, Tregs are emerging as an attractive therapeutic strategy against neurodegenerative diseases [14, 15]. Therefore, adoptive cell therapy using Tregs has attracted attention as an individualized medicine for inflammatory diseases [16]. Treg cell therapy has been attempted in mouse models of neurodegenerative diseases such as amyotrophic lateral sclerosis (ALS) and Parkinson’s disease (PD) to evaluate its neuroprotective effects [17, 18]. In our previous study, we adoptively transferred Tregs into 3×Tg-AD mice containing three mutations associated with familial Alzheimer’s disease (APP Swedish, MAPT P301L, and PSEN1 M146V) and demonstrated the inhibitory effects of Tregs on the accumulation of amyloid-beta (Aβ) and activation of microglia in the hippocampus [19]. Since TMT-treated animal models are used to study hippocampus-specific neurodegeneration that accompanies microglial activation, similar to that seen in AD, we aimed to confirm that Treg cell therapy is also effective in TMT-induced hippocampal neurodegeneration. Based on the effects of Treg therapy in other neurodegenerative diseases, it is expected that TMT-induced neuronal loss and behavior disorders will prevent through microglial activation by Treg transfer.

There is some evidence indicating that antigen-specific Tregs may be more efficient, so the generation and expansion of antigen-specific Tregs are important in Treg cell therapy [20]. To generate antigen-specific Tregs, we presented fibrillar Aβ to bone marrow-derived dendritic cells (Aβ-DCs) and performed ex vivo Treg expansion in the presence of Aβ-DCs. In addition, to increase the efficiency of Treg expansion, we treated cells with bee venom phospholipase A2 (bvPLA2), a Treg expansion inducer [21]. We previously demonstrated that bvPLA2 induced the Treg population by suppressing apoptosis [22]. Moreover, we reported that administration of bvPLA2 had neuroprotective effects on AD and PD mouse model [23, 24].

In the present study, we attempted to expand Aβ-specific Tregs and examine the effects of the adoptive transfer of these Tregs on behavioral deficits, memory formation, and neuronal loss in TMT-induced neurodegenerative mice. Furthermore, we sought to determine whether the effects of Tregs are associated with microglial activation, which induces pro-inflammatory responses. Our findings would be helpful in developing a new treatment strategy for neurodegenerative diseases.

## Materials and methods

### Animals

Seven-week-old male C57BL/6 mice were purchased from Taconic Farms, Inc. (Samtako Bio Korea, Kyunggi, Korea) and Deahan Biolink (Chungbuk, Korea). The mice were maintained under a 12-h light/dark cycle and temperature-controlled conditions, with food and water ad libitum. All experiments were performed in accordance with the approved animal protocols and guidelines established by Kyung Hee University (KHUAP(SE)-18-073).

### Regulatory T cell preparation

To prepare fibrillary Aβ, 5 mM Aβ1–42 peptide (Genscript, NJ, USA) in dimethyl sulfoxide (DMSO) was diluted with 10 mM HCl to a final concentration of 100 µM Aβ and incubated overnight (O/N) at 37 °C. Bone marrow (BM)-leukocytes from femurs and tibiae of mice were resuspended in a medium containing 20 ng/mL granulocyte-macrophage colony-stimulating factor (GM-CSF; R&D Systems, Minneapolis, MN, USA) [25]. After 7 days, BM-leukocytes were washed with magnetic-activated cell sorting buffer (Miltenyi Biotec Inc., CA, USA) and dendritic cells (DCs) were isolated using CD11c^+^ MicroBeads (Miltenyi). The DCs were resuspended at a density of 2 × 10^5^/mL and seeded in 96-well U-bottom plates. For antigen presentation, DCs were treated with 0.5 µM fibrillated Aβ for 24 h. CD4^+^ T cells from splenocytes were isolated using CD4 (L3T4) MicroBeads (Miltenyi), resuspended at a density of 2 × 10^6^/mL, and added to the DC culture at a ratio of 10: 1 (CD4^+^ T cells: DCs) with 0.4 µg/mL bvPLA2 (Sigma-Aldrich, MO, USA). Four days after CD4 T cell–DC co-culture, CD4^+^CD25^+^ T cells (Tregs) were isolated using MACS, according to the manufacturer’s protocol (CD4^+^CD25^+^ Regulatory T Cell Isolation Kit; Miltenyi). CD4^+^CD25^+^ regulatory T cells were stimulated using the Treg Expansion Kit (Miltenyi) for 2 weeks. To confirm the purity of isolated cells and the change in phenotype, cells were stained with fluorescently labeled antibodies and analyzed using flow cytometry. The following antibodies were used (1:1000): PE-CD11c (eBioscience, San Diego, CA, USA) for DC purity, PE-CD4 (BD Pharmingen, CA, USA) for CD4 T cell purity, and PE-CD127 (eBioscience), PE-Cy7-CD4 (Invitrogen, CA, USA), APC-CD62L (Invitrogen), and APC-Cy7-CD25 (BD Pharmingen) for Treg phenotype. Samples were washed with the BD FACS stain buffer (BD Bioscience, CA, USA) and stained for 30 min at 4 °C in the dark. After staining, the cells were washed 2 times with the stain buffer. The data were acquired using a BD FACSlyric™ flow cytometer (BD Bioscience) and analyzed using BD FACSuite software (BD Bioscience).

### BV2 microglia and Treg co-culture

BV2 microglia were incubated at 37 °C with 95% humidity and 5% CO_2_ for all experiments. To examine the effects of Tregs on microglial polarization, 1 × 10^6^ BV2 microglia in Dulbecco’s modified Eagle’s medium (DMEM; Welgene Daegu, Korea) 500 µL were seeded into 12-well plates. After 2–3 h, Tregs were co-cultured with BV2 cells (BV2:Treg = 10:1) and the cells were immediately stimulated with 3 µM TMT for 24 h according to previous study [26]. The cell culture supernatants were collected for ELISA, and the remaining adherent cells were harvested for mRNA extraction.

### Animal experiments

For TMT (Sigma-Aldrich, Steinheim, Germany) treatment, the mice were intraperitoneally (i.p.) administered TMT (2.6 mg/kg) and randomly divided into five groups of 18 to 25 mice, except for the control group (*n* = 21) that did not receive TMT. After 7 days, Treg cells (4 × 10^4^, 2 × 10^5^, or 1 × 10^6^) were intravenously injected (i.v.) into the tail vein of TMT-treated mice. Aricept (3 mg/kg; Eisai Co. Ltd, Tokyo, Japan) was orally administered once daily for 2 weeks from day 7.

### Behavior tests

Ten days after Treg injection, spatial learning and memory were examined in mice using the Morris water maze (MWM) test with minor modifications [27]. The water maze was a circular pool with a 90-cm diameter and was filled with opaque water containing 1 kg of powdered skim milk (maintained at 22 ± 2 °C). During training, a 6-cm hidden platform was fixed 1 cm below the water surface. The pool was surrounded by different extra-maze cues. The maximal trial duration was 60 s, with 30 s on the platform at the end of the first trial. Each animal was trained for one of the different starting positions and swimming paths once per day for 4 days. All mice were subjected to three trials per day at intervals of 15 min for 4 consecutive days. For the probe trial, the platform was removed from the pool, and the mice were allowed to swim freely for 60 s to search for the previous location of the platform. Escape latency, time spent in the platform quadrant, and the number of platform crossings were recorded for each mouse.

The elevated plus maze (EPM) test was performed after the first MWM training to measure the anxiety levels in mice. The EPM equipment was a cross-shaped maze that was elevated to a height of 50 cm above the floor. It consisted of two opposite open arms and two closed arms. Mice were positioned on the central platform and allowed to explore the maze for 3 min.

Data were collected using a video camera connected to a video recorder and a tracking device (S-MART, Pan-Lab).

### Immunohistochemistry

After the behavioral test, mice were anesthetized by pentobarbital (50 mg/kg, i.p.) and transcardially perfused with formalin and PBS. The brain was transferred into a 30% sucrose solution, and frozen-sectioned on a sliding microtome into 30-μm-thick coronal sections. The brain sections (3–5 sections/mice) were washed with phosphate-buffered saline (PBS) and incubated for 10 min with 3% hydrogen peroxide (Sigma-Aldrich) to quench endogenous peroxidase activity. Nonspecific binding was reduced by blocking the sections with 1.5% bovine serum albumin (BSA; Millipore, MA, USA) in PBS for 1 h. The sections were incubated with antibodies (1:500) for mouse CREB (Cell Signaling Technology, MA, USA), Iba1 (WAKO, Osaka, Japan), PKC (Abcam, MA, USA), NeuN (Abcam), or NGF (Invitrogen) for 24 h at RT. Brain sections were washed with PBS, incubated with a biotinylated secondary antibody (Vectastain ABC kit; Vector Laboratories, CA, USA) for 2 h, and processed using an avidin–biotin peroxidase complex kit (Vectastain ABC kit; Vector Laboratories) for 1 h. Each marker was visualized by incubation with 0.05% diaminobenzidine–HCl (DAB; Vector Laboratories). The labeled sections were mounted and analyzed under a bright-field microscope (Nikon) and the intensities were quantified using the ImageJ software (US National Institutes of Health; available at http://rsb.info.nih.gov/ij/) as previously describe [28, 29]. Data were analyzed under the same conditions by two observers for each experiment in blinded conditions to avoid the bias. Images were calibrated into an array of 512 × 512 pixels corresponding to a tissue area. Each pixel resolution had 256 Gy levels, and the intensity of immunoreactivity was evaluated based on the ROD, which was obtained after transformation of the mean gray level using the following formula: ROD = log_10_ (256/mean gray level).

### RT-PCR assay

Mice were transcardially perfused with PBS after anesthetization. RNA was isolated from the brain and BV2 cells using the easy-BLUE RNA extraction kit (iNtRON Biotechnology, Seoul, Korea), and cDNA was synthesized using Cyclescript reverse transcriptase (Bioneer, Seoul, Korea). The samples were prepared for real-time PCR using the SensiFAST SYBR no-Rox kit (Bioline, OH, USA). Real-time quantitative PCR was performed using CFX Connect (Bio-Rad, WA, USA) and the data were analyzed using CFX Maestro Software (Bio-Rad). The amplification conditions were 95 °C for 30 s, followed by 50 cycles at 95 °C for 10 s and 55 °C for 30 s. The expression levels of each target mRNAs, 2^−dCt^ values, were normalized to those of mouse β-actin, a housekeeping gene used as an endogenous control [30]. Then the relative mRNA expression values were calculated as a fold change in which the mean value of the control group considered 1. The base sequences of the primers are shown in Table 1.

**Table 1:** The base sequence of primers for rtPCR

Primer name	Forward primer sequence (5′-3′)	Reverse primer sequence (5′-3′)
β-actin	GTG CTA TGT TGC TCT AGA CTT CG	ATG CCA CAG GAT TCC ATA CC
NOS2	CAG CTG GGC TGT ACA AAC CTT	CAT TGG AAG TGA AGC GTT TCG
IL-1β	AAG CCT CGT GCT GTC GGA CC	TGA GGC CCA AGG CCA CAG G
IL-6	TTC CAT CCA GTT GCC TTC TTG	GGG AGT GGT ATC CTC TGT GAA GTC
TNFα	GGC AGG TTC TGT CCC TTT CAC	TTC TGT GCT CAT GGT GTC TTT TCT
TGFβ	GAG GTC ACC CGC GTG CTA	TGT GTG AGA TGT CTT TGG TTT TCT C
BDNF	GGA ATT CGA GTG ATG ACC ATC CTT TTC CTT AC	CGG ATC CCT ATC TTC CCC TTT TAA TGG TCA GTG
Mrc1	TTC GGT GGA CTG TGG ACG AGC	ATA AGC CAC CTG CCA CTC CGG
Ym1	TGG AGG ATG GAA GTT TGG AC	GAG TAG CAG CCT TGG AAT GT
Arg1	CTC CAA GCC AAA GTC CTT AGA G	AGG AGC TGT CAT TAG GGA CAT C

### ELISA

After anesthetization, mice were transcardially perfused with PBS. Total protein was isolated from the brain using RIPA buffer (Biosesang, Seoul, Korea) with protease and phosphatase inhibitors (Thermo Fisher Scientific, CA, USA). Levels of pro-inflammatory cytokines were quantified using TNF-α, IL-1β, and IL-6 DuoSet ELISA (R&D Systems) and normalized to the levels of BSA. The cytokines secreted by BV2 cells were measured using BV2 cell culture media and TNFα and TGFβ DuoSet ELISA (R&D Systems). The optical density was measured at 450 nm using a microplate reader (Versamax Microplate Reader, USA). All fold changes were expressed relative to those in the control group.

### Live cell imaging

CDr20 is a microglia-specific biofluorescence probe with high performance for visualizing live microglia both in vitro and in vivo [31]. For time-lapse imaging, 5 × 10^4^ BV2 microglia in 1 mL were seeded into 4-well chambers and cultured for 2–3 h before live-cell imaging was performed. Approximately 5 × 10^4^ mouse Tregs were seeded onto each chamber containing microglia. Microglia were continuously observed from pre-activation to post-activation with TMT (3 µM) treatment in the presence of 0.5 mM CDr20 (1 µM) every 3 min for a total of 30 min under the red fluorescent channel (excitation at 570 nm and emission at 600 nm). The change in the region of intensity (ROI) of each cell was measured for 30 min. All observations were performed using a DeltaVision imaging system (GE, Boston, MA, USA). To assess the intensity of fluorescence live-cell imaging, the SoftWorX software (v.6.1.3, GE) was used. CDr20 was kindly provided by Dr. YT Chang (Pohang University of Science and Technology, Pohang, Korea).

### Statistical analysis

All data were analyzed using GraphPad Prism 5.01 (GraphPad Software Inc., CA, USA). The data are presented as the mean and standard error of the mean (SEM) where indicated. All statistical significance of each variable was evaluated by one-way analysis of variance (ANOVA), followed by Tukey multiple comparison test for multiple comparisons except the intensity of PKC and time-lapse live imaging: *p < 0.05, **p < 0.01, ***p < 0.001. The intensity of PKC and time-lapse live imaging were analyzed using two-tailed Student’s *t*-test and two-way ANOVA followed by Bonferroni post-tests, respectively. All experiments were performed in a blinded manner and repeated independently under identical conditions.

## Results

### Isolation and ex vivo expansion of Tregs

**Fig. 1:**
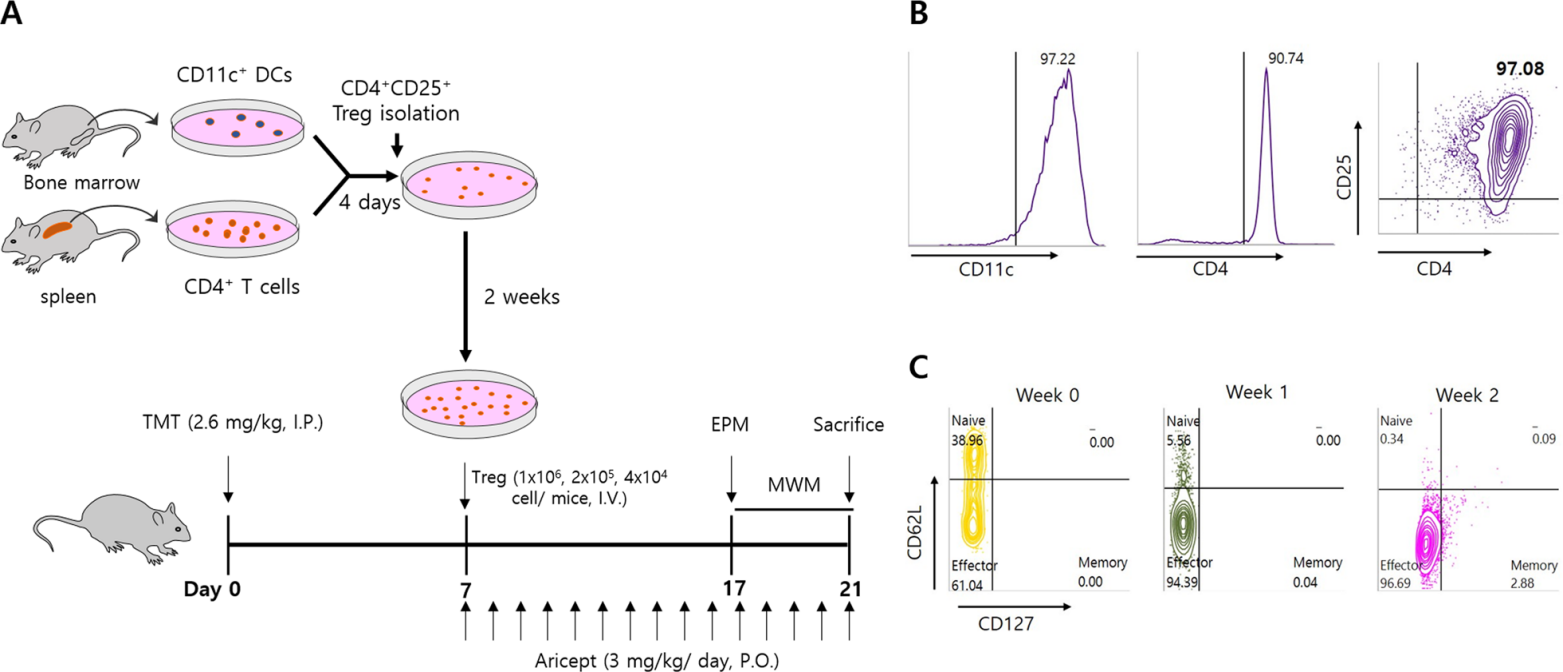
Isolation and ex vivo expansion of Tregs. CD11c^+^ dendritic cells and CD4^+^ T cells were isolated from bone marrow leukocytes and splenocytes, respectively. CD4^+^CD25^+^ Tregs were isolated after 4 days of CD11c^+^ DC and CD4^+^ T cell co-culture and expanded for 2 weeks. For the in vivo study, TMT was injected into all groups except the non-treated control group. The TMT group consisted of only TMT-intoxicated mice. Aricept group was treated with Aricept as a positive control. For the Treg group, 4 × 10^4^, 2 × 10^5^, or 1 × 10^6^ expanded Tregs were injected per mouse. After the behavioral test, mice were killed (**A**). The purity (**B**) and phenotype (**C**) of the isolated cells were analyzed by flow cytometry

To prepare Aβ-specific Tregs, CD4^+^CD25^+^ Tregs were isolated after 4 days of co-culture of CD11c^+^ DCs and CD4^+^ T cells. After ex vivo expansion, Tregs were injected into TMT-intoxicated mice (Fig. 1A). The isolated cells were analyzed using flow cytometry. FACS analysis demonstrated that CD11c^+^ DCs and CD4^+^ T cells were more than 90% enriched for these subsets. The purity of CD4^+^CD25^+^ Tregs was greater than 97% (Fig. 1B). During expansion, the changes in phenotypes were analyzed for 2 weeks. Various subsets depend on the phenotype of Tregs. For example, CD62L is highly expressed in the naïve phenotype, and CD127, the IL-7 receptor α chain, is considered a memory marker [32, 33]. Some studies have suggested the importance of the Treg phenotype, especially the CD62L^+^ naïve phenotype, for clinical manipulation. The CD62L^+^ Treg subset is an optimal suppressor that expands far more easily in culture [34]. Therefore, the phenotypes of Tregs were divided into CD62L^hi^CD127^low^ naïve, CD62L^low^CD127^low^ effector, and CD62L^low^CD127^hi^ memory phenotypes (Fig. 1C). At week 2, the transferred Tregs were mainly effector phenotypes (96.69%).

### Regulatory T cells prevent cognitive impairments in TMT-intoxicated mice

**Fig. 2:**
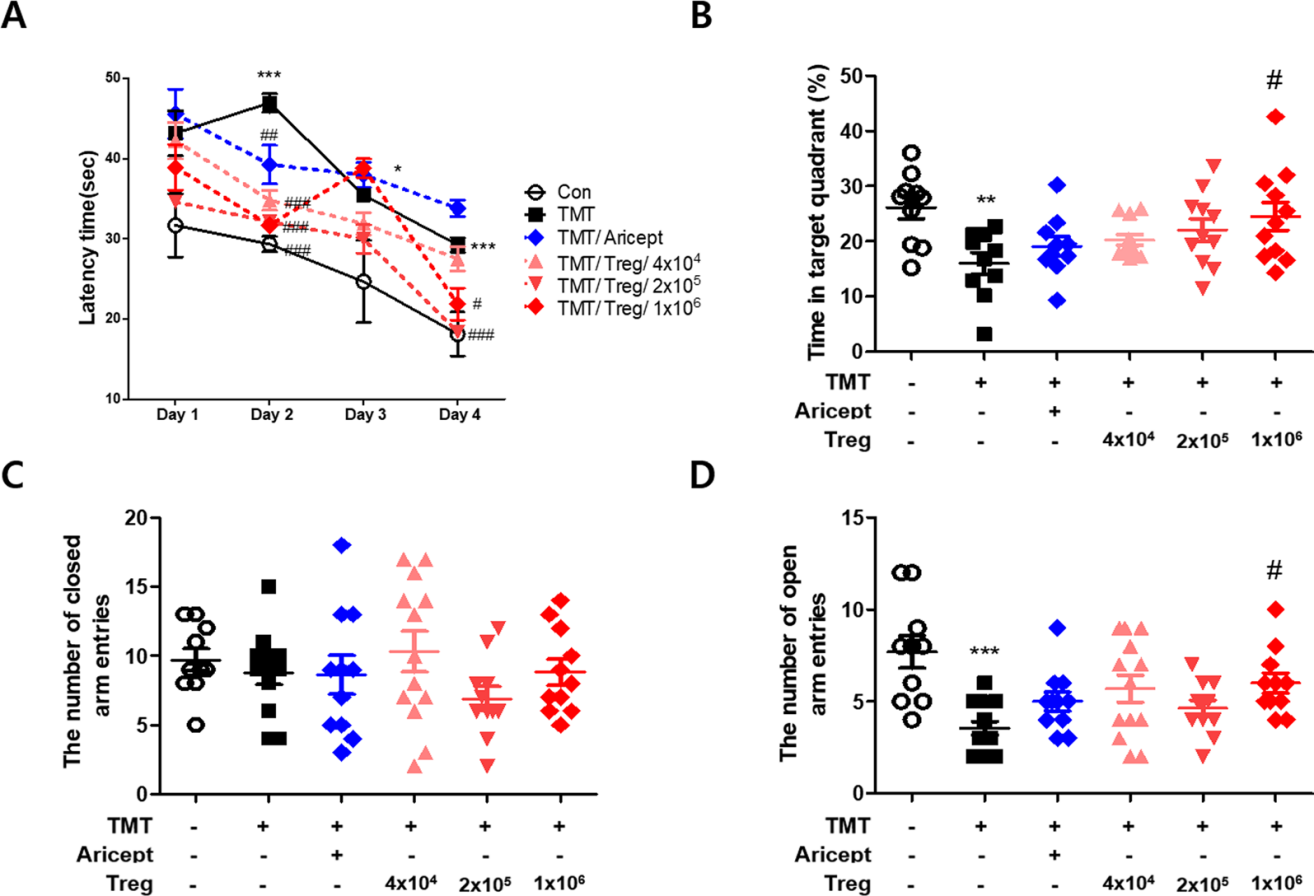
Treg improved the behavioral disorder of TMT-intoxicated mice. The MWM test was performed 10 days after Treg injection. Latency time (s) on a hidden platform (**A**) and time in quadrant (%) (**B**) were measured (*n* = 10–13 mice/group). The EPM test was performed 10 days after Treg injection. Number of closed arm entries (**C**) and number of open arm entries (**D**) were recorded (*n* = 10–13 mice/group). Error bars represent the mean ± SEM. Significance was determined by Tukey’s HSD test (*p < 0.05, ***p < 0.001 vs. the Con group and ^#^*p* < 0.05, ^##^*p* < 0.01, ^###^*p* < 0.001 vs. the TMT group)

To measure the effect of regulatory T cell transfer on spatial learning and memory ability in TMT-induced mice, the MWM test was conducted. TMT–intoxicated mice exhibited longer latency times than control mice on days 2, 3, and 4. However, the latency times of Treg groups (2 × 10^5^, 1 × 10^6^) were decreased compared to those of the TMT group (Fig. 2A). The time spent in the target quadrant was also significantly increased in the 2 × 10^5^ and 1 × 10^6^ Treg groups compared with those in the TMT group (Fig. 2B). The elevated plus maze test was used to measure anxiety in TMT-intoxicated mice. The number of entries into the closed or open arms was recorded. The number of open arm entries in the TMT group was significantly decreased compared with that of the control group, whereas that of the 1 × 10^6^ Treg group was significantly increased compared with that of the TMT group (Fig. 2C, D). These data indicate that adoptive transfer of Tregs reverts cognitive deficits in TMT-intoxicated mice.

### Regulatory T cells improve synaptic strengthening and memory function in TMT- intoxicated mice

**Fig. 3:**
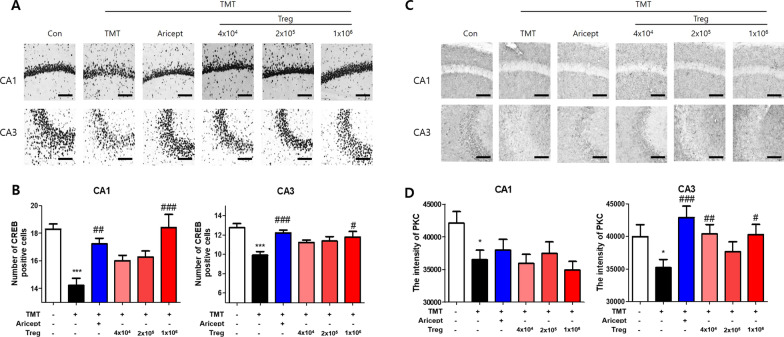
Treg increased the expression of CREB and PKC in the brain of TMT-intoxicated mice. Immunohistochemistry was performed for CREB expression in the hippocampi of TMT-intoxicated mice (**A**). The number of CREB-positive cells among CA1 and CA3 (**B**) cells was measured (*n* = 10–13 mice/group). PKC expression was assessed in the hippocampi (**C**), and the intensity of PKC in CA1 and CA3 (**D**) was measured using ImageJ software (*n* = 5–8 mice/group). Data are presented as the mean ± SEM. Significance was determined by Tukey’s HSD and *t*-test (*p < 0.05, ***p < 0.001 vs. the Con group and ^#^*p* < 0.05, ^##^*p* < 0.01, ^###^*p* < 0.001 vs. the TMT group)

CREB immunostaining was performed on sections of the mouse brain (Fig. 3A). Remarkable losses of CREB-positive cells in both CA1 and CA3 were observed in the TMT group compared to the control group. However, adoptive transfer of 1 × 10^6^ Tregs resulted in a significantly greater number of CREB-positive cells (Fig. 3B). The expression of PKC, an upstream protein kinase that activates CREB, was measured in the CA1 and CA3 regions (Fig. 3C). TMT intoxication reduced the intensity of PKC in both CA1 and CA3 compared to that in the control (Fig. 3D). The intensity of PKC in CA1 was not altered significantly following adoptive transfer of Tregs; however, adoptive transfer of Tregs increased the intensity of PKC in CA3, especially at doses of 4 × 10^4^ and 1 × 10^6^ cells/mouse.

### Tregs inhibit neuronal loss in TMT-intoxicated mice

**Fig. 4:**
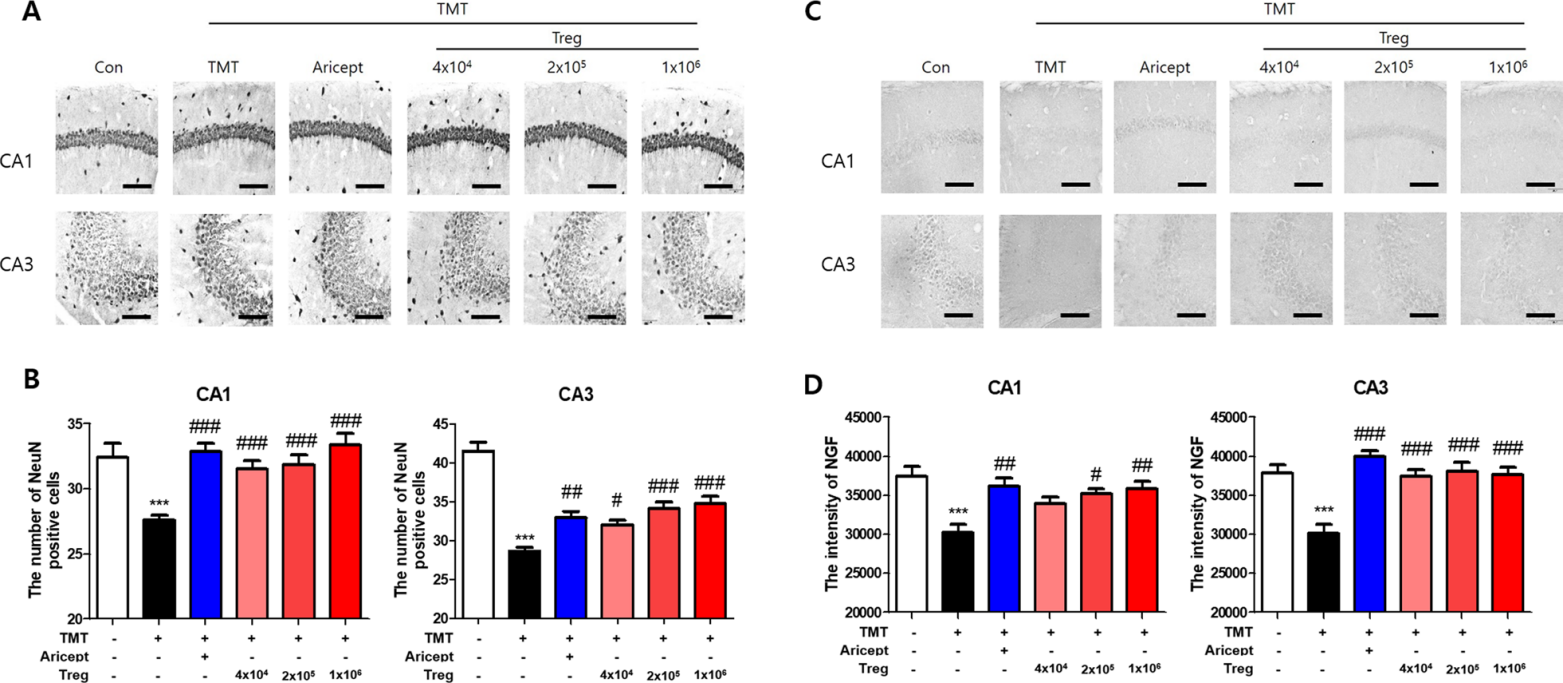
Treg increased the expression of NeuN and NGF in the brain of TMT-intoxicated mice. Immunohistochemistry was performed for NeuN expression in the hippocampi of TMT-intoxicated AD mice (**A**). The number of NeuN-positive cells among CA1 and CA3 cells (**B**) was measured (*n* = 5–8 mice/group). Immunohistochemistry was performed for NGF expression in the hippocampi of TMT-intoxicated AD mice (**C**). The intensity of NGF-positive cells in CA1 and CA3 (**D**) was measured (*n* = 5–8 mice/group). Data are presented as the mean ± SEM. Significance was determined by Tukey’s HSD (***p < 0.001 vs. the Con group and ^#^*p* < 0.05, ^##^*p* < 0.01, ^###^*p* < 0.001 vs. the TMT group)

To evaluate the effect of Tregs on TMT-induced neuronal loss, mouse brain sections were stained for NeuN, a neuronal biomarker (Fig. 4A). The number of NeuN-positive cells was significantly reduced in TMT-treated mice compared with that in the control in both CA1 and CA3 (Fig. 4B). All groups adoptively transferred Tregs showed more NeuN-positive cells than the TMT group. Additionally, the expression of NGF, a neurotrophic factor, was assessed (Fig. 4C). Similar to that for NeuN, the intensity of NGF staining that was significantly decreased in CA3 upon TMT intoxication was recovered upon Treg transfer (Fig. 4D). These results suggest that adoptive transfer of Tregs inhibits TMT-induced neuronal loss.

### Tregs reduce pro-inflammatory microglial activation in TMT-intoxicated mice

**Fig. 5:**
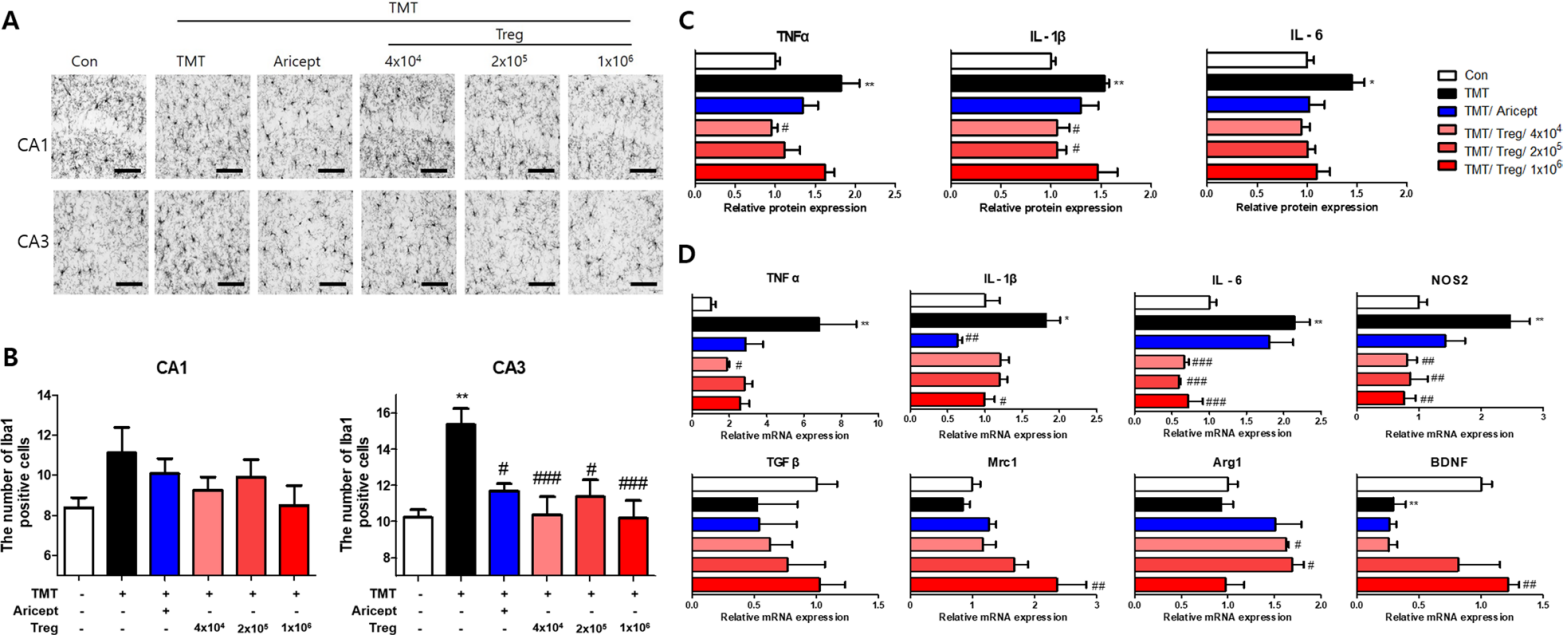
Treg inhibited pro-inflammatory factors via microglial activation in the brain of TMT-intoxicated. The expression of Iba1 was observed in the hippocampi of TMT-intoxicated mice using immunostaining (**A**). The number of Iba1-positive cells in CA1 and CA3 (**B**) was measured (*n* = 10–13 mice/group). The protein levels of pro-inflammatory cytokines, including TNFα, IL-1β, and IL-6, in the brain were measured using ELISA and calculated as a relative for Con (**C**) (*n* = 3–5 mice/group). The relative mRNA levels of pro-inflammatory microglia-associated markers, including TNFα, NOS2, IL-1β, and IL-6, and anti-inflammatory markers, TGFβ, Mrc1, Arg1, and BDNF in the brain were analyzed (**D**) (*n* = 3–5 mice/group). Data are presented as the mean ± SEM. Significance was determined by Tukey’s HSD (*p < 0.05, **p < 0.01 vs. the Con group and ^#^*p* < 0.05, ^##^*p* < 0.01, ^###^*p* < 0.001 vs. the TMT group)

To assess microglial activation, mouse brain sections were stained for Iba1, an activated microglial marker (Fig. 5A). The number of Iba1-positive cells in the TMT group was significantly increased compared with that in the control group, whereas that in all Treg-transfer groups was significantly decreased compared with that in the TMT group in CA3. Similar tendencies were observed in CA1, but the difference was not significant (Fig. 5B). The protein levels of pro-inflammatory cytokines, such as TNFα, IL-1β, and IL-6, were measured using ELISA (Fig. 5C). The expression of these cytokines was significantly increased in TMT-intoxicated mice compared with that in the control. When Tregs were adoptively transferred, the levels of these cytokines showed decreasing tendencies compared with those in the TMT group. Next, mRNA levels in the brain were measured using real-time PCR (Fig. 5D). The mRNA levels of pro-inflammatory microglial markers TNFα, IL-1β, IL-6, and NOS2 in the TMT group were significantly increased compared with those in the control group. In all Treg-transfer groups, the mRNA levels of NOS2 and IL-6 were significantly decreased compared with those in the TMT group. The mRNA levels of TNFα and IL-1β showed similar trends. However, the mRNA levels of TGFβ, Mrc1, Arg1, and BDNF showed tendencies opposite to those shown by the pro-inflammatory microglial markers. Collectively, the results indicate that adoptive transfer of Tregs inhibits TMT-induced microglial activation, especially that of pro-inflammatory M1 microglia.

### Tregs induce transition of M1–M2 phenotypes in TMT-treated microglia

**Fig. 6:**
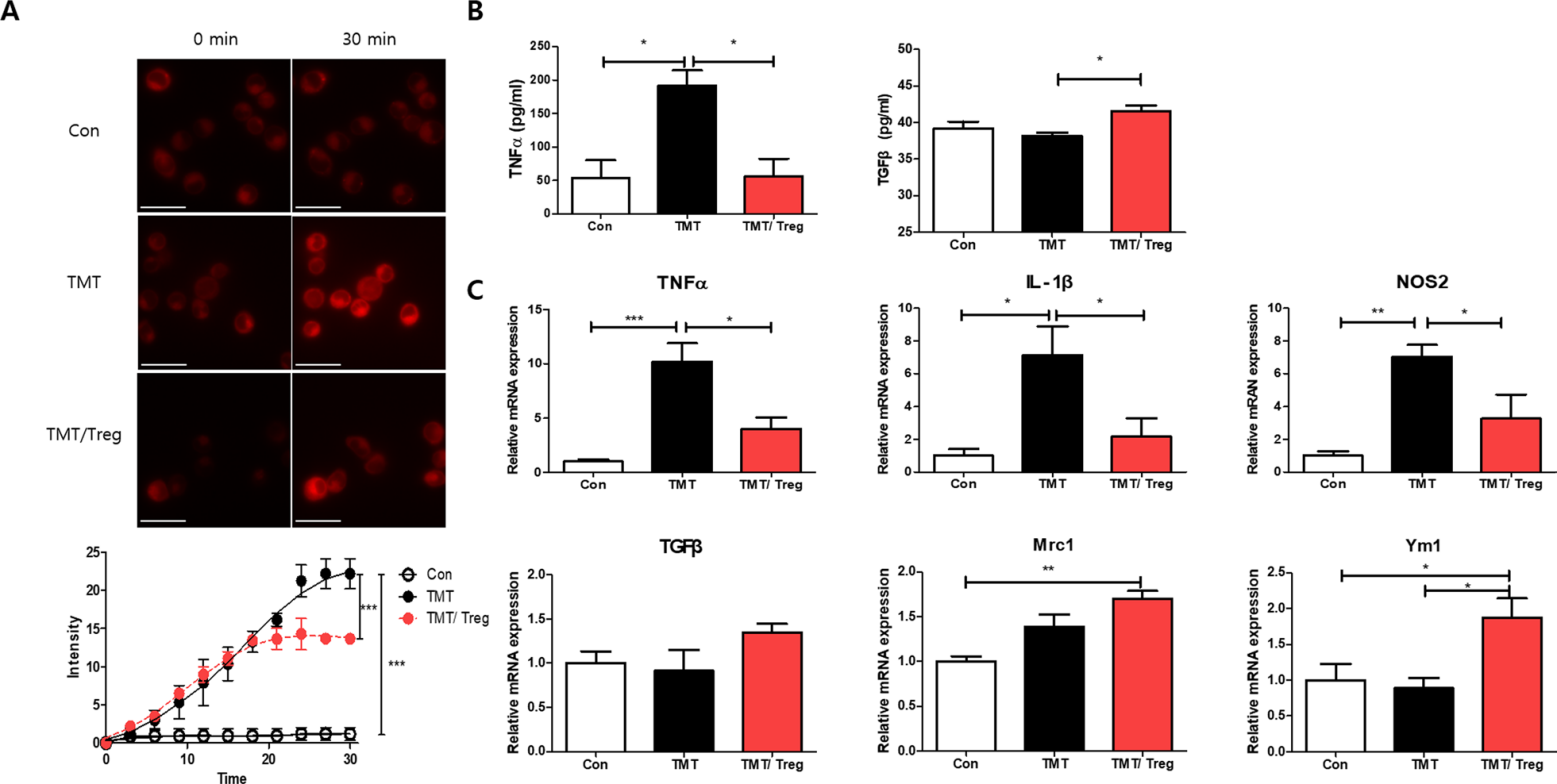
Treg modulates TMT-induced microglial activation and polarization. Time-lapse live imaging of BV2 microglial cells was monitored for 30 min with a CDr20 live microglia-specific probe (**A**). Representative images show microglial activity at 0 and 30 min with TMT treatment in the presence of CDr20 under the red fluorescent channel (excitation at 570 nm and emission at 600 nm). BV2 microglial cells were co-cultured with Tregs and stimulated with TMT for 24 h. The secreted levels of TNF-α and TGF-β (**B**) were measured using ELISA with supernatants. Relative mRNA expression of M1 cytokines, TNF-α and IL-1β; M1 microglial maker, NOS2; M2 cytokine TGFβ; M2 microglial makers, Mrc1 and Ym1 was examined and normalized to actin (**C**). Data are presented as the mean ± SEM. Significance was determined by Bonferroni’s correction and Tukey’s HSD (*p < 0.05, **p < 0.01, ***p < 0.001). Scale bar = 50 μm

To confirm the direct impact of Tregs on microglial activation, we utilized murine BV2 microglia under TMT-induced inflammatory conditions in vitro. For live imaging, live BV2 cells were imaged using CDr20, a high-performance fluorogenic chemical probe for activated microglia [31], during which, the intensity of the labeled cells was recorded for 30 min (Fig. 6A). As BV2 cells were activated upon TMT treatment, the intensity gradually increased for 30 min. However, co-culture with Tregs showed inhibitory effects on the TMT-induced activation of microglia. To further confirm the effect of Tregs on microglial polarization, the release of the pro-inflammatory cytokine TNFα and anti-inflammatory cytokine TGFβ was measured using ELISA (Fig. 6B). TNFα levels were higher in TMT-treated BV2 cells than in normal BV2 cells. Furthermore, in co-culture with Tregs, the level of TNFα significantly decreased, whereas that of TGFβ increased compared with that in TMT-treated BV2 cells. The mRNA expression of the M1 microglial markers TNFα, NOS2, and IL-1β and M2 microglial markers TGFβ, Mrc1, and Ym1 were also measured (Fig. 6C). As expected, the levels of the pro-inflammatory M1 microglial markers were increased in TMT-treated BV2 cells, and co-culture with Tregs substantially decreased the mRNA expression of these markers. Conversely, the expression of the M2 microglial markers increased upon co-culture with Tregs. Taken together, these data suggest that Tregs modulate microglial polarization upon TMT treatment.

## Discussion

In this study, we investigated the effects of Tregs on TMT-induced hippocampal neurodegeneration. We found that Tregs not only improved cognitive function, but also reduced anxiety in TMT-intoxicated mice. Moreover, Tregs inhibited neuronal loss, and the neuroprotective effects of Tregs could potentially be attributed to suppression of microglia-mediated neuroinflammation. Compared with Aricept, a drug used for AD, adoptive transfer of Tregs was found to be similarly or more effective. Our study supports the potential of Treg therapy for hippocampal neurodegeneration.

Tregs are considered attractive therapeutic targets for attenuating inflammation. Tregs play roles in inhibiting pro-inflammatory cytokines and inducing neurotrophic factors and apoptosis of pro-inflammatory microglia, ultimately promoting neuroprotection [35]. In a previous study from our laboratory, adoptive transfer of Tregs was attempted in 3×Tg-AD mice, upon which a clear delay in the onset of AD neuropathology was observed. In addition, the neuroprotective effect of Tregs was demonstrated, including reduction in Aβ deposition and microglial activation in the hippocampus. However, Treg adoptive transfer has never been attempted in the TMT-induced neurodegenerative model, although it has been considered as a model of AD-like disease in rats [36]. Therefore, in this study, we transplanted Tregs into TMT-intoxicated mice to alleviate TMT-induced hippocampal neurodegeneration.

For clinical application, various strategies were proposed to improve the effects of Treg therapy. The most common method is developing antigen-specific Tregs instead polyclonal Tregs which may lead to off-target suppression. Antigen presentation could enhance the therapeutic utility of T cell transfer to induce target sites [37, 38]. Aβ is also present in the normal brain; however, it is misfolded and deposited in the hippocampus in several pathological conditions such as AD. Therefore, it is regarded as one of the characteristics of these diseases [39]. Moreover, since Aβ accumulation was detected in TMT-intoxicated mice, we chose Aβ as an antigen for presentation to adoptively transfer Tregs [40]. Additionally, we treated bvPLA2 during antigen presentation to expand the Treg population. It was reported that bvPLA2 induces Treg population both in vivo and in vitro and significantly suppresses apoptosis in Tregs [21, 22]. The combination of antigen presentation via DCs and bvPLA2 treatment for the generation and expansion of Aβ-specific Tregs is an important attempt of this study. The effects and mechanism of action of Aβ presentation and bvPLA2 treatment on the efficacy of Tregs remain unclear. This will be investigated in a future study.

For decades, TMT-induced neurodegenerative models, especially rats and mice, have been used as good research tools. In the rat model, TMT administration induces a progressive cell death accompanied by microglial activation in CA1 and CA3 like AD [36, 41]. Notably, TMT injection into mice can also cause dentate gyrus (DG) granular cell apoptosis. Many studies on TMT-induced mouse model focused on neuropathology in DG [42–46]. DG is the site where adult hippocampus neurogenesis occurs and most information of DG is sent to CA3 to CA1 according to the tri-synaptic pathway in the hippocampus [47]. Some studies reported neuronal self-repair following TMT-induced neuronal loss in DG. These data indicated that neuronal regeneration occurs in DG approximately 7–10 days after TMT intoxication [48–51]. Therefore, TMT has been considered as a toxicant that acts exclusively on DG in mice. However, there are several reports that TMT induced neurodegeneration was not restricted in DG. KR Reuhl and colleagues reported extensive degenerative and necrotic changes in CA3 after TMT intoxication [52]. In another report, IB4, a microglial marker, and Fas, an apoptotic molecule, were increased in CA1 after TMT intoxication [53]. Nevertheless, degenerative change in neurons of CA was unremarkable than DG, TMT has been considered as a neurotoxicant that selectively affects in DG [54]. However, studies on TMT-induced cognitive dysfunction have emerged [55, 56]. In recent studies, it was reported that memory impairment accompanied by neurodegeneration in CA induced by TMT intoxication in mice. According to these studies, neuronal loss was observed in CA1 even after 7 days of TMT intoxication, unlike in DG [57–60]. This implied that the timing of TMT-induced neurotoxicity on DG and CA is different, probably it occurs later in CA than in DG. It is a very interesting topic that these events could affect the tri-synaptic circuit related disorders and will be revealed in further studies. In the present study, we focus on TMT-induced cognitive disorders and molecular change in CA1 and CA3.

It is reported that TMT-intoxicated animals developed cognitive impairment and hyperactivity [7]. The results of MWM and EPM test showed that high dose of Tregs improved these behavior changes. But Aricept, a positive control, showed no significant effect. It is probably because Aricept is not an effective drug for long-term treatment. In fact, it has been reported that Aricept treatment for 16 weeks did not improve cognitive function in APPswe/PS1dE9 mice [61]. These results imply that Treg possess a sufficient potential as a more effective treatment option than Aricept.

CREB is a key molecule in synaptic strengthening, memory formation, and neurogenesis. It controls the transcription of genes involved in neuronal growth and survival and the lack of CREB gene results in neurodegeneration. Indeed, disruption of the CREB phosphorylation mechanism results in a reduction in CREB activation following memory impairment in AD [62–64]. Likewise, TMT-induced memory impairment was observed upon inhibition of CREB activation and was alleviated by regulation of the CREB-signaling pathway in the hippocampus [65]. Since CREB plays a critical role in short- to long-term memory, drugs targeting CREB itself have been proposed for memory modification [66]. One of the molecules present the upstream of CREB and regulating it is PKC. Therefore, activation of PKC leads to CREB phosphorylation [67, 68]. In addition, PKC itself performs neurogenesis-related functions, including cell differentiation and proliferation and immune-related processes. In a previous study, *Bacopa monnieri* (L.) Wettst. extract prevented TMT-induced hippocampal damage via PKC [69]. NGF is also a neurotrophic factor that enhances neurogenesis [70]. We showed that TMT intoxication induced neuronal cell death, represented by the expression of NeuN, in both CA1 and CA3, whereas there was no difference in DG (data not shown). These results are consistent with the possibility of different TMT toxicity timing on DG and CA mentioned above. And Tregs, especially at high dosage, increased the expression of CREB, PKC, and NGF as well as NeuN. This suggests that adoptively transferred Tregs not only prevent neuronal loss, but also induce neurogenesis in the hippocampus.

Microglia are phagocytic macrophages that comprise 10–15% of the total cells in the CNS. Since they can be either beneficial or harmful depending on their activation status, their polarization is considered a potential therapeutic target in neurodegenerative diseases such as AD. Classically activated “M1” microglia contribute to inflammation by secreting free radicals, NOS2, and pro-inflammatory cytokines such as IL-1, IL-6, and TNFα. Neuroinflammation amplifies microglial activation and further worsens the disease. In contrast, alternatively activated “M2” microglia promote tissue repair by releasing neuroprotective cytokines such as IL-10, TGFβ, and IGF1. Therefore, microglial polarization is considered an attractive therapeutic strategy against cognitive disorders [71, 72]. Indeed, there have been many studies that treat neurodegenerative diseases by shifting microglial phenotypes. Some studies reported behavior recovery following enhancing M2 microglia in not only AD, but also traumatic brain injury and spinal cord injury [73–75]. It is well known that pro-inflammatory microglial activation and cytokine secretion are associated with TMT intoxication [76–79]. Therefore, we confirmed the activation and polarization of microglia in vivo and in vitro. As expected, microglial activation and pro-inflammatory marker expression were increased upon TMT intoxication. However, Tregs inhibited the activation of M1 but enhanced M2 microglia in vivo. Additionally, we observed microglial activation over time using time-lapse live imaging in vitro. TMT treatment activated microglia for 30 min, but co-culture with Tregs suppressed this activation. ELISA and RT-PCR data showed that this inhibition by Tregs targeted M1 microglia. Based on these in vivo and in vitro results, Tregs could effectively inhibit microglial activations and covert microglial phenotype from M1 to M2. These changes in microglia phenotype lead to neurogenesis and ultimately improve cognitive impairment. It is in line with those of previous studies showing that Tregs modulate microglia and alleviate neurodegenerative disorders [11, 72, 80, 81].

## Conclusions

**Fig. 7:**
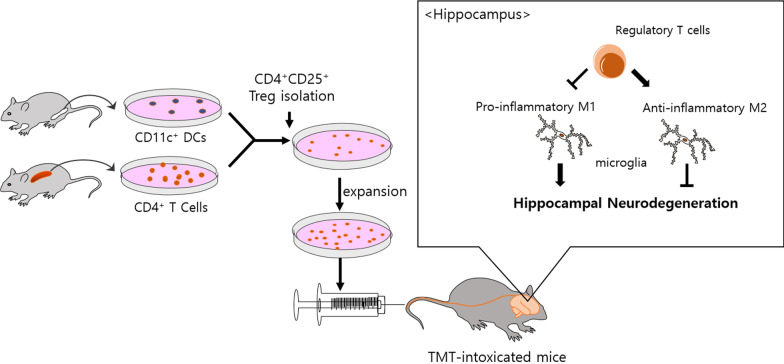
Treg has neuroprotective effects by modulating microglial polarization in TMT-intoxicated mice. Ex vivo expanded and adoptively transferred Tregs ameliorates hippocampal neurodegeneration in TMT-intoxicated mice. Tregs promotes microglial phenotype shift from pro-inflammatory M1 to anti-inflammatory M2, resulting in a neuroprotective effects on behavioral deficits, memory formation, neuronal loss, and neuroinflammation

Taken together, as shown in Fig. 7, we report that adoptive transfer of Tregs reduces behavioral deficits in TMT-intoxicated mice. Tregs inhibit neuronal loss and increase the expression of factors that enhance neurogenesis. In particular, Tregs dramatically modulated the activation and polarization of microglia upon TMT intoxication both in vivo and in vitro. These findings support the potential of Treg therapy in hippocampal neurodegeneration.

## Data Availability

Not applicable.

## Abbreviations

Aβ: Amyloid-β
AD: Alzheimer’s disease
ALS: Amyotrophic lateral sclerosis
Arg1: Arginase 1
BDNF: Brain-derived neurotrophic factor
BM: Bone marrow
BSA: Bovine serum albumin
bvPLA2: Bee venom phospholipase A2
CNS: Central nervous system
CREB: CAMP response element-binding protein
DAB: Diaminobenzidine
DCs: Dendritic cells
DG: Dentate gyrus
DMSO: Dimethyl sulfoxide
EPM: Elevated plus maze
GM-CSF: Granulocyte-macrophage colony-stimulating factor
Iba1: Ionized calcium binding adaptor molecule 1
IL: Interleukin
Mrc1: Mannose receptor 1
MWM: Morris water maze
NOS2: Nitric oxide synthase 2
NeuN: Neuronal nuclear protein
NGF: Nerve growth factor
PBS: Phosphate-buffered saline
PD: Parkinson’s disease
PKC: Protein kinase C
TGFβ: Transforming growth factor β
TMT: Trimethyltin
Tregs: Regulatory T cells
TNFα: Tumor necrosis factor α
